# Development of a New Method for Calculating Intraocular Lens Power after Myopic Laser In Situ Keratomileusis by Combining the Anterior–Posterior Ratio of the Corneal Radius of the Curvature with the Double-K Method

**DOI:** 10.3390/jcm11030522

**Published:** 2022-01-20

**Authors:** Yoshihiko Iida, Kimiya Shimizu, Nobuyuki Shoji

**Affiliations:** 1 Department of Ophthalmology, Kitasato University School of Medicine, Sagamihara 252-0374, Japan; nshoji@kitasato-u.ac.jp; 2 Eye Center, Sanno Hospital, Tokyo 107-0052, Japan; kimiyas@iuhw.ac.jp

**Keywords:** IOL power calculation after LASIK, no-history method, cataract surgery, anterior–posterior ratio of the corneal radius of the curvature

## Abstract

Background: A new method, the Iida–Shimizu–Shoji (ISS) method, is proposed for calculating intraocular lens (IOL) power that combines the anterior–posterior ratio of the corneal radius of the curvature after laser in situ keratomileusis (LASIK) and to compare the predictability of the method with that of other IOL formulas after LASIK. Methods: The estimated corneal power before LASIK (Kpre) in the double-K method was 43.86 D according to the American Society of Cataract and Refractive Surgery calculator, and the K readings of the IOL master were used as the K values after LASIK (Kpost). The factor for correcting the target refractive value (correcting factor [C-factor]) was calculated from the correlation between the anterior–posterior ratio of the corneal radius of the curvature and the refractive error obtained using this method for 30 eyes of 30 patients. Results: Fifty-nine eyes of 59 patients were included. The mean values of the numerical and absolute prediction errors obtained using the ISS method were −0.02 ± 0.45 diopter (D) and 0.35 ± 0.27 D, respectively. The prediction errors using the ISS method were within ±0.25, ±0.50, and ±1.00 D in 49.2%, 76.3%, and 96.6% of the eyes, respectively. The predictability of the ISS method was comparable to or better than some of the other formulas. Conclusions: The ISS method is useful for calculating the IOL power in eyes treated with cataract surgery after LASIK.

## 1. Introduction

The opportunities to perform cataract surgery in patients who have undergone corneal refractive surgeries such as laser in situ keratomileusis (LASIK) are increasing. The problem in performing cataract surgery after corneal refractive surgery is the incorrect IOL power calculation. Especially, the result of the IOL power calculation after myopic corneal refractive surgery causes a hyperopic shift [1,2,3,4,5]. The reason for residual hyperopia is inaccurate measurement of K value after corneal refractive surgery and incorrect effective lens position (ELP) calculated using third-generation theoretical formulas in which the post-corneal refractive surgery K value that was flattened is used [6,7]. A number of theoretical and empirical approaches have been proposed to solve this problem [1,2,3,4,5,7,8,9,10,11,12,13,14,15,16,17,18,19,20,21,22,23,24,25,26,27].

Among the approaches, the double-K method, described by Arramberri [7] in 2003, enables more accurate IOL power calculation by estimating the ELP using pre-refractive surgery corneal measurements (Kpre) and the subjective refractive value-derived K values (clinical history method) as Kpost for optical calculations without using the post-refractive surgery corneal measurements. This principle makes sense; however, many patients unfortunately do not have the necessary data for the clinical history method, such as Kpre and subjective refractive values before and after refractive surgery.

Corneal refractive power measurements are inaccurate in the post-refractive surgery eye, because the assumption of estimating total corneal refractive power from the radius of the curvature of the corneal anterior surface, an algorithm used by keratometers and topographers, is not valid. The reason is the change in the relationship between the anterior and posterior corneal radii of the curvature, which is no longer 7.5/6.3 [8]. This invalidates the value of the different corneal indexes of refraction (standardized index of refraction = 1.3375), which allows total corneal power calculation from the anterior surface radius of the curvature in nonoperated eyes [9,10]. The Scheimpflug anterior segment imaging system (Pentacam, Oculus GmbH) can measure the posterior corneal radii. Excimer laser ablation thins the corneal thickness and flattens the curvature plane of the anterior cornea, which changes the corneal refractive power. In the case of excimer laser ablation, the greater the amount of correction and anterior–posterior ratio of the corneal radius, the greater the error in the K value.

In cases where the double-K method is performed (in which we do not have pre-refractive surgery data), the prediction error is expected to depend on the anterior–posterior ratio of the corneal radius when using postoperative keratometric K values for the double-K method. This study devised a new no-history method, the Iida–Shimizu–Shoji (ISS) method, based on the double-K method using the prediction error induced by the ratio of the anterior and posterior radii of the corneal curvature after LASIK. This method can calculate IOL frequencies without relying on preoperative data. The accuracy of this new method was compared with other formulas in eyes after LASIK.

## 2. Materials and Methods

### 2.1. Patients and Methods

This study included Japanese patients who underwent cataract surgery after LASIK at the Department of Ophthalmology, Kitasato University Hospital. This retrospective review of the data was approved by the Institutional Review Board at the Kitasato University (B17-292) and conformed to the Declaration of Helsinki.

### 2.2. Cataract Surgical Procedures

For the cataract surgery, standard phacoemulsification was performed using topical anesthesia. The surgical technique consisted of capsulorhexis, nucleus, and cortex extractions, as well as IOL implantation, through a 2.8 mm temporal clear corneal incision, in all the cases. Nontoric monofocal IOLs (AQ-110NV, STAAR Surgical, Chiba, Japan) were implanted. All the surgeries were uneventfully performed by two experienced surgeons (Y.I. and K.S.) using the same technique.

### 2.3. Estimation of Refractive Error in the Double-K Method Induced by the Anterior–Posterior Ratio of the Corneal Radii

To establish a method for estimating the refractive error in the double-K method induced by the anterior–posterior ratio of the corneal radii, we retrospectively studied 30 eyes of 30 consecutive patients who underwent cataract surgery after LASIK for myopia. Only one type of IOL (AQ-110NV) was used for each eligible case; one eye was used per patient; and, for cases in which surgery was performed on both eyes, the eye of the previously operated eye was included. All patients did not have relevant historical data. Table 1 shows the patient parameters.

The axial length (L) and K readings were measured with the IOL master partial coherence interferometer device (Carl Zeiss Meditec, Jena, Germany) in all of the cases. Corneal topography using the Pentacam Scheimpflug system was performed before cataract surgery for each patient. The ratio between the anterior and posterior radii of the corneal curvature, which were the averages of the central radii of the steep and flat meridians in the 3.0 mm zone measured with the Pentacam Scheimpflug system, respectively, was defined as the anterior–posterior (A–P) ratio. The estimated corneal power before corneal refractive surgery (Kpre) in the double-K method was 43.86 D according to the American Society of Cataract and Refractive Surgery (ASCRS) calculator (https://iolcalc.ascrs.org/, accessed on 20 June 2021), and the K readings of the IOL master were used as the K value after corneal refractive sur-gery (Kpost). The IOL power calculation used the double-K method based on the SRK/T formula with Kpre and Kpost, as mentioned earlier. The double-K method was calculated by entering the data into a spreadsheet software (Microsoft Excel):

(1) $$\begin{array}{l} \;Predicted\;postoperative\;refraction\\ =\frac{1000n_{a}\left(n_{a}r_{post}-\left(n_{c}-1\right)\mathrm{LOPT}\right)-\mathrm{LP}\left(LOPT-ACD_{est}\right)\left(n_{a}r_{post}-\left(n_{c}-1\right)ACD_{est}\right)}{n_{a}\left(V\left(n_{a}r_{post}-\left(n_{c}-1\right)LOPT\right)+LOPTr_{post}\right)-0.001LP\left(LOPT-ACD_{est}\right)\left(V\left(n_{a}r_{post}-\left(n_{c}-1\right)ACD_{est}\right)+ACD_{est}r_{post}\right)} \end{array}$$

where *na* is the refractive index of intraocular media (1.336), *rpost* is the radius of curvature of the anterior corneal surface measured by IOL master (*rpost* = 337.5/Kpost), *nc* is the refractive index of the cornea (1.333), *LOPT* is the adjusted axial length considering that is measured with optical biometry (L + 0.65696 − 0.02029 L), *LP* is the implanted IOL power, and *ACDest* is the ELP (named *ACDest* in the original publication [6,7]) of the double-K method. *ACDest* is calculated from corneal height in mm and Offset, where the corneal height is calculated using Kpre (43.86D); offset is calculated from A constants.

**Figure 1:**
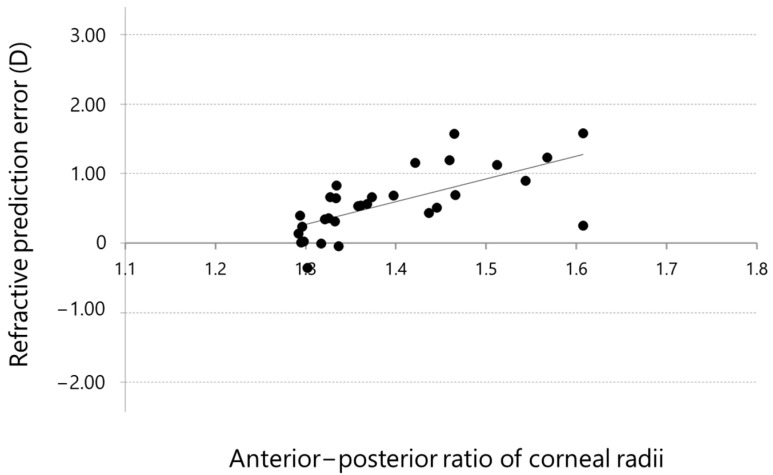
Correlation between the anterior–posterior ratio of corneal radii and refractive prediction error (Pearson correlation coefficient, R = 0.678, *p* < 0.001).

The prediction errors were calculated by subtracting the predicted postoperative refraction from the postoperative manifest refraction (spherical equivalent) 1 month after cataract surgery. The prediction error and A–P ratio were plotted on a scattergram (Figure 1).

The two parameters were significantly correlated (Pearson correlation coefficient, R = 0.678, *p* < 0.001), and the best-fit regression equation was obtained as follows:

*y* = 3.28 *x* − 4.00. (2)

The predicted refraction error obtained from this regression equation was defined as the correction factor (C-factor):

C-factor = 3.28 × A–P ratio − 4.00.(3)

The ISS method corrects the target refraction value of the double-K method based on the SRK/T formula by adding the C-factor (*C*). The predicted refraction by the ISS method is expressed as follows:

(4) $$\begin{array}{c} Predicted\;postoperative\;refraction\;by\;the\;ISS\;method\;\\ =\frac{1000n_{a}\left(n_{a}r_{post}-\left(n_{c}-1\right)\;LOPT\;\right)-\;LP\;\left(\;LOPT\;-\;ACD{\;}_{est\;}\right)\left(n_{a}r_{post}-\left(n_{c}-1\right)\;ACD{\;}_{est\;}\right)}{n_{a}\left(V\left(n_{a}r_{post}-\left(n_{c}-1\right)LOPT\right)+LOPTr_{post}\right)-0.001LP\left(LOPT-ACD_{est}\right)\left(V\left(n_{a}r_{post}-\left(n_{C}-1\right)ACD_{est}\right)+ACD_{est}r_{post}\right)}+C \end{array}$$

The *IOL* power for the desired refraction using the ISS method is obtained as follows:

(5) $$\begin{array}{c} Lpower\;for\;the\;desired\;refraction\;using\;the\;ISS\;method\;\\ =\frac{1000n_{a}(n_{a}r_{post}-\left(n_{c}-1\right)LOPT-0.001(DR-C)\left(V\left(n_{a}r_{post}-\left(n_{C}-1\right)LOPT\right)+\;LOPT\;r_{post}\right)}{\left(LOPT-ACD_{est}\right)(n_{a}r_{post}-\left(n_{c}-1\right)ACD_{est}-0.001(DR-C)\left(V\left(n_{a}r_{post}-\left(n_{c}-1\right)ACD_{est}\right)+ACD_{est}r_{post}\right)} \end{array}$$

where *DR* is the desired refraction after cataract surgery, *C* is the C-factor, and *V* is the vertex distance (12 mm).

### 2.4. Intraocular Lens Power Calculations Using the ISS Method and Comparison of the Predictability of the ISS Method with That of Other Formulas or Methods

Fifty-nine eyes of 59 consecutive patients in another group who underwent cataract surgery after the group studied in item 2.3 were included in the study. Table 2 shows the patient parameters. The IOL power was calculated using the ISS method. The postoperative refraction (manifest refraction) was obtained 1 month after cataract surgery.

The predictability of the ISS method was compared with the Shammas no-history method [15,22], Haigis-L formula [24], Potvin–Hill pentacam method [25], and Barrett True K no-history formula [28,29]. These IOL calculation formulas or methods do not require preoperative data and were performed using the ASCRS IOL power calculator.

The prediction refractive error was calculated from the difference between the actual postoperative manifest refraction and the predicted refraction for each formula or method. The mean numerical error; median absolute error; mean absolute error; and percentages of eyes within ±0.25, ±0.50, and ±1.00 D of the target refraction were compared among formulas and methods.

### 2.5. Statistical Analyses

We conducted statistical analyses using commercially available statistical software (BellCurve for Excel, Social Survey Research Information Co., Ltd., Tokyo, Japan). The relationship between two sets of data was analyzed using Pearson correlation coefficient test. The one-sample t-test was used to assess whether the mean numerical refraction prediction errors produced by the various methods were significantly different from zero. The Wilcoxon signed-rank test was used to compare the absolute predicted refractive error with the ISS-method and with the other formulas. The percentages of eyes within ±0.25, ±0.50, and ±1.00 D of the target correction were compared with the ISS method and other calculation methods using the Fisher exact test. The Bonferroni correction was applied for multiple tests. The results were expressed as mean ± standard deviation, and values of *p* < 0.05 were considered statistically significant.

## 3. Results

The mean values of the numerical and absolute prediction errors using the ISS method were −0.02 ± 0.45 D (range, −1.11 to 0.96 D) and 0.35 ± 0.27 D (range, 0.01 to 1.11 D), respectively. The median value of the absolute prediction errors was 0.29 D. The prediction errors using the ISS method were within ±0.25 D in 29 eyes (49.2%), ±0.50 D in 45 eyes (76.3%), and ±1.00 D in 57 eyes (96.6%).

**Figure 2:**
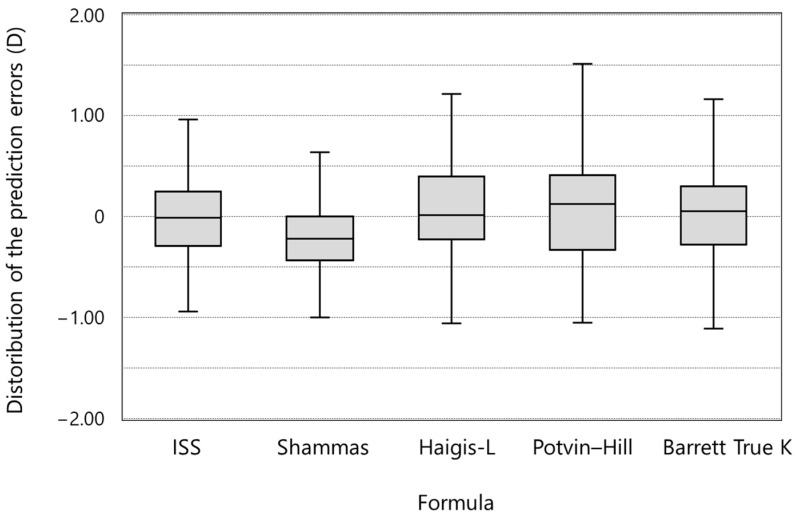
Intraocular lens power prediction errors using various methods (ISS = Iida–Shimizu–Shoji method; Shammas = Shammas no-history method; Haigis-L = Haigis-L formula; Potvin–Hill = Potvin–Hill pentacam method; Barrett True K = Barrett True K ho-history formula).

Figure 2 shows the distributions of the prediction errors. Table 3 shows the numerical and absolute prediction errors of the targeted refraction retrospectively when various formulas were used. In terms of numerical prediction errors, the Shammas no-history method showed a statistically significant difference from zero and myopic shift. In terms of median absolute error, the ISS method median absolute error was significantly lower than those of Shammas no-history method (*p* = 0.028) and Potvin–Hill pentacam methods (*p* = 0.025).

**Figure 3:**
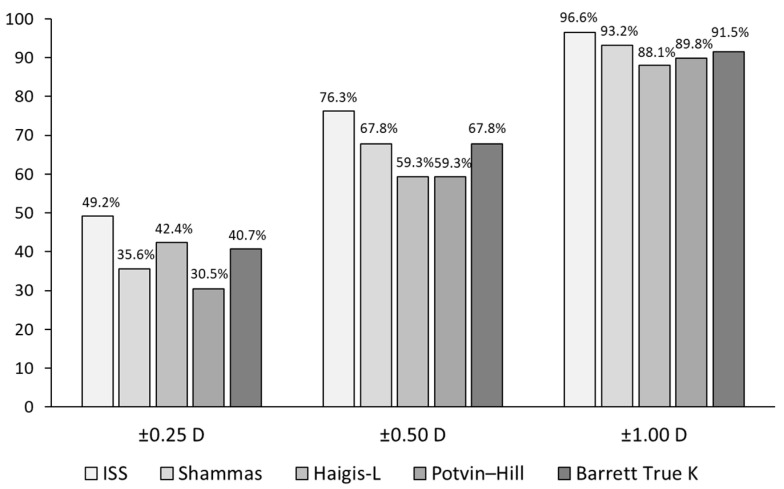
Comparison of the percentages of eyes within ±0.25, ±0.50, and ±1.00 D from the target refraction between IOL power calculation formulas (ISS = Iida–Shimizu–Shoji method; Shammas = Shammas no-history method; Haigis-L = Haigis-L formula; Potvin–Hill = Potvin–Hill pentacam method; Barrett True K = Barrett True K no-history formula).

Figure 3 shows the percentages of eyes within ±0.25, ±0.50, and ±1.00 D from the target refraction. There were no statistically significant differences between the groups.

## 4. Discussion

In this study, we examined the predictability of the ISS method, a no-history method we devised, and the formula for post-refractive surgery that can be calculated with the ASCRS calculator. The predictability of the ISS method was not only comparable to that of other formulas, but also better than that of some other formulas.

The principle of the ISS method is simple: the predicted refractive value is calculated using the C-factor, which is calculated from the correlation between the refractive error obtained by the double-K method and the A–P ratio of the radius of curvature of the cornea.

Preoperative data are required for Kpre, which is used in the double-K method, and Kpost must be obtained using the clinical history method, but if preoperative data are sometimes not available, the calculation cannot be performed. When selecting preoperative and postoperative data, care should be taken to select the timing of the data. Refractive changes not only in the cornea, but also in the lens may be included in the data. Wang et al. reported that the no-history method had better predictability than the method using preoperative data or changes in refractive values before and after refractive surgery [30].

Each of the no-history methods available in the ASCRS calculator has the following characteristics: the Shammas no-history method uses regression analysis to estimate corneal power after LASIK/PRK by adjusting the K measurement (Kpost), and the Kpost is the average K value from the IOLMaster. The Shammas-PL formula is used for IOL power calculation [15,22]. The Haigis-L formula takes the corneal radius measured by the IOLMaster and generates a corrected corneal radius using the Haigis-L algorithm, which is then used in the normal Haigis formula to calculate the IOL power after myopic laser vision correction [24]. The Potvin–Hill pentacam method uses regression analysis to estimate corneal power after LASIK/PRK using the TNP_Apex_Zone40 values from pentacam and values for ocular axial length and anterior chamber depth (if available). This method uses the Shammas-PL formula to calculate IOL power [25]. The Barrett True-K formula uses the Universal II formula, which is a modification of the original Universal Theory formula [28]. The other is the “True-K no-history formula”, which is calculated only from data obtained when the patient undergoes cataract surgery. Details regarding the design of the True-K and Universal II formulas have not been released.

In the present study, the numeric refractive prediction error of the Shammas no-history method was significantly far from 0 D and myopic shifted, and the absolute refractive prediction error was significantly larger in the Shammas no-history method and Potvin–Hill pentacam methods than in the ISS method. As the Shammas-PL formula was used to calculate IOL power in the Potvin–Hill pentacam method, it is possible that these two formulas caused the significant difference.

In comparison with the figures in the literature, in 104 eyes with previous LASIK, Wang et al. reported that the median absolute refractive prediction error of 0.39 D for Haigis-L, 0.48 D for Shammas, and 0.42 D for Barrett True-K [31]. In 246 eyes with previous LASIK/PRK, Ianchulev et al. reported a median absolute refractive error of 0.53 D for Haigis-L and 0.51 D for Shammas [32]. In 58 eyes with previous LASIK/PRK, Abulafia et al. reported a median absolute refractive error of 0.46 D for Shammas, 0.58 D for Haigis-L, and 0.33 D for Barrett True-K [29]. Our study showed better results than these reports, but this difference is probably due to differences in the population groups studied and whether or not the type of IOL was standardized.

Although the formula is not included in the ASCRS calculator, as a calculation method using the same pentacam and using the anterior–posterior surface of the corneal radius of curvature, Saiki et al. focused on the fact that the posterior corneal surface data did not change before and after excimer laser corneal refractive surgery and developed the posterior corneal curvature radius; they developed the A–P method to estimate Kpre before corneal refractive surgery based on the radius [26,27]. However, even if the preoperative K value can be predicted, as long as the third-generation IOL power formula involving K value is used to calculate ELP, it is known that the use of K value to calculate ELP can be one of the causes of refractive error even in cases without refractive surgery [33], so refractive may contain at least the same amount of refractive error factors as cataract cases that have not undergone surgery.

The ISS method uses a constant value of 43.86 D as Kpre, which is used in the ASCRS calculator. Although accurate prediction of ELP is difficult, setting Kpre to a constant value in the ISS method reduces the effect on the K value on the refractive error characteristic of the third-generation IOL power calculation formula, because the K value is involved in the calculation of ELP, and this may be one of the reasons the refractive error becomes smaller.

There are limitations to this study. The main limitation is the small sample size. The C factor used in the ISS method was calculated using a regression equation, but only 30 eyes were subject to this regression equation for post-LASIK cataract surgery cases. However, we believe that we were able to eliminate the influence of internal correlation by targeting one eye per case and that of refractive error by the type of IOL by limiting to one type of IOL. In addition, by increasing the number of cases in the regression equation in the future, we may be able to adjust the C-factor and further improve the prediction accuracy. The number of cases in the group that compared the ISS method with the other methods was 59 eyes, but they were consecutive cases that were completely different from the cases used in the regression equation for determining the C-factor; in addition, like the cases in the regression equation, they were limited to one person, one eye, and one type of IOL.

In conclusion, multiple calculation methods are available for calculating the IOL power after refractive surgery, and it is necessary to select an IOL by calculating from multiple options. The no-history method can be calculated in all the cases, including those with preoperative data, and the ISS method can be useful for calculating the IOL power in eyes that have undergone cataract surgery after LASIK.

## Figures and Tables

**Table 1:** Parameters of patients who underwent cataract surgery after corneal refractive surgery used for obtaining the regression formula to estimate the C-factor of the ISS method.

Parameter	Post-LASIK (*n* = 30)
	Mean ± SD (Range)
Age (years)	55.4 ± 10.3 (22–71)
Axial length (mm)	26.75 ± 1.67 (24.81–29.63)
Mean K by IOL master (D) Mean corneal radius of curvature by IOL master (mm) (keratometric index = 1.3375)	38.90 ± 2.35 (33.08–41.88) 8.68 ± 0.56 (8.06–10.20)
Mean anterior corneal radius of curvature by Pentacam (mm)	8.73 ± 0.58 (7.97–10.45)
Mean posterior corneal radius of curvature by Pentacam (mm)	6.33 ± 0.26 (5.71–6.88)

K = keratometric readings; D = diopter; LASIK = laser in situ keratomileusis.

**Table 2:** Parameters in patients who underwent cataract surgery after LASIK in comparing the predictability of the various formulas (*n* = 59).

	Mean ± SD (Range)
Age (years)	59.0 ± 9.3 (36–77)
Axial length (mm)	27.01 ± 1.94 (23.99–32.76)
Mean K by IOL master (D) Mean corneal radius of curvature by IOL master (mm)(keratometric index = 1.3375)	38.95 ± 2.54 (33.84–43.25) 8.66 ± 0.57 (7.80–9.97)
Mean anterior corneal radius of curvature by Pentacam (mm)	8.68 ± 0.55 (7.81–9.86)
Mean posterior corneal radius of curvature by Pentacam (mm)	6.36 ± 0.29 (5.70–7.31)
TNP (4.0 mm) by Pentacam (D)	37.30 ± 2.55 (31.60–41.80)

K = keratometric readings; D = diopter; LASIK = laser in situ keratomileusis, TNP = true net power.

**Table 3:** The refractive prediction error of the targeted refraction using the various formulas.

Formula/Method	Refractive Prediction Error (D)				
	Numerical		Absolute		
	Mean ± SD (Range)	*p*-Value	Mean ± SD (Range)	Median	*p*-Value vs. ISS
ISS	−0.02 ± 0.45	0.770	0.35 ± 0.27	0.29	N/A
	(−1.11–0.96)		(0.01–1.11)		
Shammas	−0.20 ± 0.54	0.005 *	0.45 ± 0.36	0.29	0.028 *
	(−1.42–1.36)		(0.00–1.42)		
Haigis-L	0.07 ± 0.59	0.361	0.45 ± 0.38	0.37	0.199
	(−1.26–1.59)		(0.00–1.59)		
Potvin–Hill	0.13 ± 0.65	0.124	0.50 ± 0.43	0.38	0.025 *
	(−1.05–2.34)		(0.02–2.34)		
Barrett True K	0.02 ± 0.58	0.754	0.43 ± 0.39	0.28	0.581
	(−1.16–1.61)		(0.03–1.61)		

* *p* < 0.05. ISS = Iida–Shimizu–Shoji method, Shammas = Shammas no-history method, Haigis-L = Haigis-L formula, Potvin–Hill = Potvin–Hill pentacam method, Barrett True K = Barrett True K no-history formula.

## Data Availability

The data presented in this study are available on request from the corresponding author.
